# Cardiac dysfunction in dialysing adults with end‐stage kidney disease is associated with exercise intolerance: A pilot observational study

**DOI:** 10.14814/phy2.70050

**Published:** 2024-09-10

**Authors:** Joe Antoun, Anthony I. Shepherd, Jo Corbett, Nicholas C. Sangala, Robert J. Lewis, Emma Lane, Zoe L. Saynor

**Affiliations:** ^1^ Physical Activity, Health and Rehabilitation Thematic Research Group, School of Sport, Health and Exercise Sciences, Faculty of Science and Health University of Portsmouth Portsmouth UK; ^2^ Academic Department of Renal Medicine, Wessex Kidney Centre Portsmouth Hospitals University NHS Trust Portsmouth UK; ^3^ Queen Alexandra Hospital Portsmouth Hospitals University NHS Trust Portsmouth UK; ^4^ School of Health Sciences, Faculty of Environmental and Life Sciences University of Southampton Southampton UK; ^5^ National Institute for Health and Care Research, Southampton Biomedical Research Centre University Hospital Southampton NHS Foundation Trust Southampton UK

**Keywords:** aerobic fitness, cardiorespiratory, chronic disease, exercise, physiology

## Abstract

People with end‐stage kidney disease (ESKD) often exhibit impaired cardiac structure and function, which may contribute to poor exercise capacity. This study used multimodal exercise testing to investigate the central and peripheral mechanisms of exercise limitation in adults with ESKD, also comparing in‐centre hemodialysis (ICHD) to home hemodialysis (HHD). Seventeen adults (55.5 ± 14.5 years; *n* = 14 male; *n* = 12 HHD) participated. Resting cardiac examinations, followed by submaximal cycling cardiopulmonary exercise testing (CPET) and functional exercise testing, revealed cardiac structural abnormalities (increased left ventricular mass) and cardiac injury. Aerobic fitness in adults with ESKD was low, with pulmonary oxygen uptake (V̇O_2_) at the gas exchange threshold (GET) occuring at 39 ± 8% predicted V̇O_2peak_. O_2_ pulse, an estimate of stroke volume (SV), was higher in HHD at rest (*p* = 0.05, *ES* = 0.58) and during unloaded cycling (*p* = 0.05, *ES* = 0.58) compared to ICHD. However, thoracic bioreactance derived SV at the GET was significantly higher in adults receiving ICHD versus HHD (*p* = 0.01, *ES* = 0.74). In adults with ESKD, cardiac output was positively associated with V̇O_2_ at the GET (*r* = 0.61, *p* = 0.04). This study highlights prevalent exercise dysfunction in adults with ESKD undergoing dialysis, with potential distinct differences between in‐centre and home hemodialysis, mechanistically linked to underlying cardiac abnormalities.

## 1 INTRODUCTION

Cardiovascular disease (CVD) is a risk factor, cause‐ and consequence of progressive kidney disease (Jankowski et al., 2021). In adults with end‐stage kidney disease (ESKD), CVD manifests as increased left ventricular (LV) load, systolic dysfunction, and vascular calcification (Jankowski et al., 2021). Cardiac dysfunction, including chronotropic incompetence (McGuire et al., 2020), reduced cardiovascular reserve (Ting et al., 2015), and cardiac injury (measured by [Troponin]) (Lankinen et al., 2021), has been implicated as a primary cause of exercise intolerance in this population, however this requires further investigation.

While changes in cardiovascular structure and function are a characteristic of ESKD (Matsuo et al., 2018), the pathogenesis of exercise (dys)function is multi‐factorial (McGuire et al., 2020; Ting et al., 2015; Wilkinson et al., 2019). Sarcopenia is prevalent (Sabatino et al., 2021) and is associated with reduced peak oxygen uptake (V̇O_2peak_) (Diesel et al., 1990) and increased mortality risk (Wilkinson et al., 2021) in adults with ESKD. There is a need to comprehensively characterize the physiological mechanism(s) underpinning exercise limitations in dialysing adults with ESKD using new techniques available, particularly comparing different treatment modalities. Home hemodialysis (HHD), with its shorter and more frequent sessions, is expected to reduce physiological burden and subsequent dysfunction, potentially leading to better maintained fitness, compared to more traditional in‐centre provision.

Cardiopulmonary exercise testing (CPET) provides comprehensive insight into exercise (dys)function and its underlying physiological mechanism(s) (Guazzi et al., 2017), but it is underutilized in people with ESKD. In earlier stages of chronic kidney disease (CKD) (McGuire et al., 2021a; Pella, Theodorakopoulou, et al., 2022; Ting et al., 2015), reduced V̇O_2peak_, a prognostically useful variable (O'Driscoll et al., 2023) in this population, and submaximal aerobic fitness (the gas exchange threshold [GET]) (McGuire et al., 2021a; Ting et al., 2015), have been reported, with the GET associated with poor echocardiography‐derived cardiac function at rest in adults with ESKD (Pella, Boutou, et al., 2022). Thoracic bioreactance, a non‐invasive cardiac output monitoring method, combined with pulmonary gas analysis and near‐infrared spectroscopy (NIRS), can further elucidate the dynamic balance between O_2_ delivery and utilization during exercise (Wilkinson et al., 2019), however, to date have been underused.

CPET provides limited information about other key fitness components, such as muscle function, gait, and balance, which are linked to falls risk, independence (Wang et al., 2017), and can independently predict all‐cause mortality in adults with ESKD (Vogt et al., 2016). Functional exercise tests, such as the short physical performance battery (SPPB; 16), 1‐minute sit‐to‐stand test (1‐minute STS (Bayoumi & Alwakeel, 2015);), gait speed (Chan et al., 2019), and peripheral muscle strength tests (Garcia et al., 2017) including handgrip dynamometry, offer complementary insights into overall fitness and the ability to perform activities of daily living (Arikan et al., 2015).

This study aimed to characterize exercise (dys)function in adults with ESKD on maintenance hemodialysis (HHD or ICHD), and provide mechanistic insights into exercise capacity in this cohort using multimodal exercise testing and measures of cardiac structure and function. We hypothesized that cardiac structure and function would be negatively associated with aerobic and functional exercise capacity in adults with ESKD, with better fitness and physiological function expected in those who dialyse at home more frequently.

## 2 METHODS

Fully informed written consent was obtained prior to enrolment into this single site, observational, cross‐sectional study in adults with ESKD. All in‐person visits were undertaken at the Wessex Kidney Centre. Participants attended a single visit (~4‐h) on a non‐dialysis day, during which all testing was performed. Venous blood samples were obtained pre‐ and post‐hemodialysis sessions, which preceded the research visit for both those receiving ICHD or HHD.

### 2.1 Participants

Dialysing adults with ESKD, receiving HHD or ICHD under the clinical care of the Wessex Kidney Centre, participated. Participants continued usual care and dialysis regimens throughout. Body mass (Marsden®, Rotherham, UK) and stature were measured to the nearest 0.01 kg and 0.01 m, respectively. Body mass index (BMI) was calculated as mass (kg) / height (m)^2^ and body surface area (BSA) estimated (Du Bois & Du Bois, 1916). Body composition and hydration status were assessed using multifrequency bioelectrical impedance analysis (BIA; Fresenius Medical Care, Bad Homburg, Germany). Disease severity and the clinical profile of each individual was determined by their nephrology consultant, using recent medical records. Sarcopenia and cachexia were characterized using the European consensus guidelines (Cruz‐Jentoft et al., 2019), based upon muscle quantity, performance and muscle strength.

### 2.2 Transthoracic echocardiography

Transthoracic echocardiography (Vivid E9, GE Healthcare, Chicago, United States) was performed at rest in accordance with standardized clinical guidelines (Echocradiography BS of. Protocols and guidelines, 2021) and interpreted by an experienced cardiac physiologist (EL). *Q̇* represents the cardiac index (CI), which was calculated as cardiac output (L·min^−1^)/body surface area (m^2^), and SV represents the SV index (SVI); (mL·m^−2^) calculated as stroke volume (mL)/body surface area (m^2^). The Simpson's biplane method of discs (Harkness et al., 2020) was used to estimate systolic function and LV volumes, subsequently used to calculate the left ventricular ejection fraction (LVEF).

LV volume in systole and diastole were subsequently expressed relative to BSA. Linear measurements of LV dimensions were also taken in the parasternal long axis in accordance with British Society of Echocardiography guidelines (Harkness et al., 2020). LV mass was calculated using these measurements and further indexed for BSA. The LV was imaged in all 3 apical windows (apical four chambers, two chambers and three chambers) and the myocardium divided using the 17‐segment model for qualitative analysis. Speckle tracking was used for assessment of longitudinal deformation in systole in echocardiographic window giving a negative value for each segment. These were further averaged to derive the global longitudinal strain (GLS), a parameter used for assessment of LV systolic dysfunction and early subclinical markers of LV systolic dysfunction (Klaeboe & Edvardsen, 2019).

### 2.3 Cardiac biomarkers

In‐centre phlebotomy was undertaken by a trained renal nurse, with all samples taken pre‐ and post‐dialysis (using a 5008S CorDiax, Fresenius, Bad Homburg, Germany) from the individual's dialysis vascular access site. For individuals dialysing at home (using a NxStage System One, Fresenius, Bad Homburg, Germany), samples were drawn either by the participant, or the individual in charge of their care (e.g. partner or family member).

Serum biomarkers of cardiac damage ([NTproBNP], [Troponin I]) and dialysis clearance ([Beta‐2‐microglobullin] ([B2M])) in adults with ESKD were assessed pre‐ and post‐hemodialysis. For those dialysis at home, 10 mL of blood was collected within serum separator tubes (SST; BD Vacutainer, BD Plymouth, United Kingdom), stored in their residential refrigerator for ≤6‐h, then returned to the hospital biochemistry department. Samples used for [Troponin I] analysis were spun at 3500 rpm for 10‐min at 4°C (Rotanta 460 centrifuge, Hettich, Tuttlingen, Germany). Samples used for [B2M] and [NTproBNP] were spun for at 3000 rpm for 5‐min within the Rotanta centrifuge and a further 4‐min at the same speed within an automated track (Power express, Beckman Coulter, California, USA) at 4°C. Samples were analyzed within 4‐h for [NT‐proBNP] and [B2M] and within 7‐h for [TnI]. Analysis for [NT‐ProBNP] was performed using enzyme‐linked fluorescent assays on a Biomerieux® Vidas (Vidas, Basingstoke, UK; CV 5.02%) and for [Troponin I] and [B2M] using enzyme‐linked immunosorbent assays on a DxI analyzer (Beckman Coulter, California, USA; CV 4.80%) and a Beckman Coulter AU680 instrument (Beckman Coulter, California, USA; CV 4.10%), respectively.

### 2.4 CPET

A submaximal ramp incremental cycling protocol on an electronically braked cycle ergometer (Corival CPET, Lode, Groningen, The Netherlands) was used. Following 3‐min of seated rest and 3‐min unloaded cycling, resistance was increased incrementally using a continuous ramp protocol by a predetermined rate (5–10 W·min^−1^), based on sex, age and PA profile. Participants were instructed to maintain a cadence between 60 and 80 revs∙min^−1^ throughout exercise, with the test terminated when ratings of perceived exertion (RPE) (Borg, 1982) exceeded 15 (“hard”). 3‐min of unloaded cycling and 10‐min seated, monitored recovery concluded CPET.

Throughout rest and exercise, breath‐by‐breath changes in pulmonary gas exchange and ventilation were measured via a face mask and turbine system (Metalyzer 3B, Cortex, Leipzig, Germany). Prior to each test, gas analyzers were calibrated using gases of known concentrations and the turbine volume transducer using a 3 L calibration syringe (Hans Rudolph, Kansas City, MO). Fingertip transcutaneous arterial O_2_ saturation (SpO_2_) was measured throughout using pulse oximetry (NONIN, Minnesota, USA) on a beat‐by‐beat basis, with exercise terminated if values dropped ≤85% or the nadir exceeded 4%. Beat‐to‐beat heart rate (HR) was measured by telemetry (H7, Polar, Kempele, Finland) and thoracic bioreactance (Cheetah Medical, Wilmington, Delaware, USA; see further details below) measured every 30‐seconds. NIRS (Artinis®, Portamon system, The Netherlands; see further details below) was also utilized to measure peripheral muscle oxygenation. Appropriate normative reference values were used for interpretation of CPET parameters (Wasserman et al., 2004).

### 2.5 Thoracic bioreactance cardiography

Throughout rest and exercise, non‐invasive thoracic bioreactance cardiography (Cheetah Medical, Wilmington, Delaware, USA), previously described and used in adults with ESKD by McGuire et al (2021b), was measured.

### 2.6 Near‐infrared spectroscopy

NIRS measurements were obtained using a commercially available continuous‐wave portable system (Artinis®, Portamon system, The Netherlands), placed on the *m. vastus lateralis*, midway between the greater trochanter and the lateral epicondyle of the femur of the dominant limb leg. This system has been described by Theodorakopoulou et al. (2023), Plastic film and dark elastic bandages (3 M®, Bracknell, United Kingdom) were used to minimize interference from extraneous light and moisture. During the pre‐exercise rest phase, the leg was standardized in the downward phase.

### 2.7 Functional tests of physical function

Balance, sit‐to‐stand ability and gait speed were assessed using the short physical performance battery (SPPB), a well‐recognized battery of tests which can assess physical function in adults with kidney disease (López‐Montes et al., 2020). The SPPB includes three different standing balance tests with different foot positions (feet together, semi‐tandem, and tandem); the sit‐to‐stand‐5 (STS5), which requires participants to complete five movements from seated to a standing position as fast as possible; and gait speed testing, measuring the time to walk 2.44 m as quickly as possible. Handgrip strength, a useful measure of nutritional and functional status in kidney disease (Wilkinson et al., 2022), was also measured. Participants completed repeated maximal handgrip strength tests, using their non‐fistula arm, with adequate rest between repetitions, until 3 measurements were within 5% (mean: 3.5 ± 0.7 repetitions).

Following this, the 1‐minute STS test was performed using a standardized protocol on a conventional chair with no arm rests (Rikli & Jones, 1999). Participants were asked to stand up and sit down as often as possible as fast as possible for 1‐minute and the number of repetitions performed recorded. Finally, hand dexterity of the non‐fistula arm side was assessed using the Moberg's picking up test (Amirjani et al., 2007), a marker of fine motor ability, both with eyes open and closed.

### 2.8 Data analysis

HR, V̇O_2_, breathing frequency (*f*_R_) carbon dioxide production (*V̇*CO_2_), minute ventilation (*V̇*_E_), and respiratory exchange ratio (RER) data were interpolated to 10‐s averages, with peak exercise values taken as the highest achieved during the incremental test. The GET was determined using the V‐slope method (Beaver et al., 1986) and verified through visual inspection of the ventilatory equivalents (*V̇*_E_/V̇O_2_ and *V̇*_E_/*V̇*CO_2_) against time and end‐tidal partial pressure for O_2_ and carbon dioxide. The arteriovenous O_2_ content difference [*C*_(a‐v)_O_2_] was estimated via the rearrangement of the Fick equation [*C*_(a‐v)_O_2_ = V̇O_2_/*Q̇*]. NIRS data were interpolated to 10‐s intervals and expressed as the change, in arbitrary units and percentage change, from baseline to end‐exercise.

### 2.9 Statistical analysis

Statistical analyses were conducted using the Statistical Package for the Social Sciences (IBM SPSS Statistics, version 27.0), with significance set a priori at *p* < 0.05 and a statistical trend toward significance set at ≤0.10. All data are expressed as mean ± standard deviation (SD) unless otherwise stated. Due to the low sample size, non‐parametric testing was undertaken; the Spearman's rank correlation coefficient was used to assess relationships between mechanistic physiological variables in the whole group and a subsequent Mann–Whitney U‐test was used to perform sub‐group analyses and compare means between those receiving HHD and ICHD. The effect size (*ES*; *r* [Rosenthal's r]) was then calculated as ‘*r* = *Z*/√*N*’, with 0.1, 0.3 and 0.5 classified as a small, moderate and large effect, respectively (Hopkins, 2002). Figures were created within Python (V3.10.2) using the matplotlib package (V3.5.1).

## 3 RESULTS

In total, 17 adults with ESKD (age: 55.5 ± 14.5 years; *n* = 14 male) participated, of which 12 were receiving HHD and 5 receiving ICHD, respectively. Participant characteristics and clinical profiles are presented in Table 1. In accordance with the revised European consensus on sarcopenia (Cruz‐Jentoft et al., 2019), 3/17 (*n* = 2 ICHD) were classified as low strength, and 8/17 (*n* = 4 ICHD) as low performance. None were classified as low muscle quantity, when assessing skeletal muscle mass through BIA. No serious adverse events occurred during this study.

**TABLE 1:** Participant characteristics and clinical profile.

	Total	ICHD	HHD	*p*‐value	ES
Age (years)	55.5 ± 14.5	55.0 ± 13.1	55.8 ± 15.6	0.79	0.06
Gender, *n* (male/female)	14/3	4/1	10/2	‐	‐
Weight (kg)	85.7 ± 20.4	97.0 ± 15.5	81.0 ± 20.9	0.14	0.36
Height (cm)	174.0 ± 8.0	173.0 ± 6.0	174.0 ± 9.0	0.53	0.15
BMI (kg·m‐^2^)	28.4 ± 7.2	32.4 ± 5.0	26.7 ± 7.4	0.04*	0.51*
BSA (m^2^)	2.02 ± 0.25	2.15 ± 0.19	1.97 ± 0.26	0.17	0.33
Body composition and hydration					
Saliva flow rate (mL·min^−1^)	0.30–0.40	0.04 ± 0.03	0.13 ± 0.20	0.69	0.11
Overhydration (L)	3.1 ± 4.2	8.5 ± 5.9	1.3 ± 1.6	0.43	0.18
Total body water (kg)	43.2 ± 5.6	42.7 ± 4.7	43.4 ± 6.2	0.87	0.05
Extracellular water (kg)	18.7 ± 3.0	18.6 ± 1.6	18.8 ± 3.6	0.69	0.11
Intracellular water (kg)	24.0 ± 6.1	22.8 ± 2.8	24.6 ± 7.2	0.96	0.02
LTM (kg)	43.2 ± 8.4	42.4 ± 8.9	43.6 ± 8.6	0.75	0.12
Hemodialysis vintage (months)	45.9 ± 43.5	53.6 ± 43.5	42.7 ± 44.9	0.34	0.23
Vascular access type (*n*)	‐	‐	‐	‐	‐
Central venous catheter	3	0	3	‐	‐
Graft	3	1	2	‐	‐
Arteriovenous fistula	11	4	7	‐	‐
Comorbidities (*n*)					
Hypertension	7	2	5	‐	‐
Diabetes	3	3	0	‐	‐
COPD	1	0	1	‐	‐
Cancer	5	0	5	‐	‐
Cardiac	6	4	2	‐	‐

Abbreviations: BMI, body mass index; BSA, body surface area; COPD, chronic obstructive pulmonary disease; HHD, home hemodialysis; ICHD, in‐centre hemodialysis; LTM, lean tissue mass.

* Denotes statistical significance between HHD and ICHD.

### 3.1 Baseline cardiovascular structure and function

**FIGURE 1:**
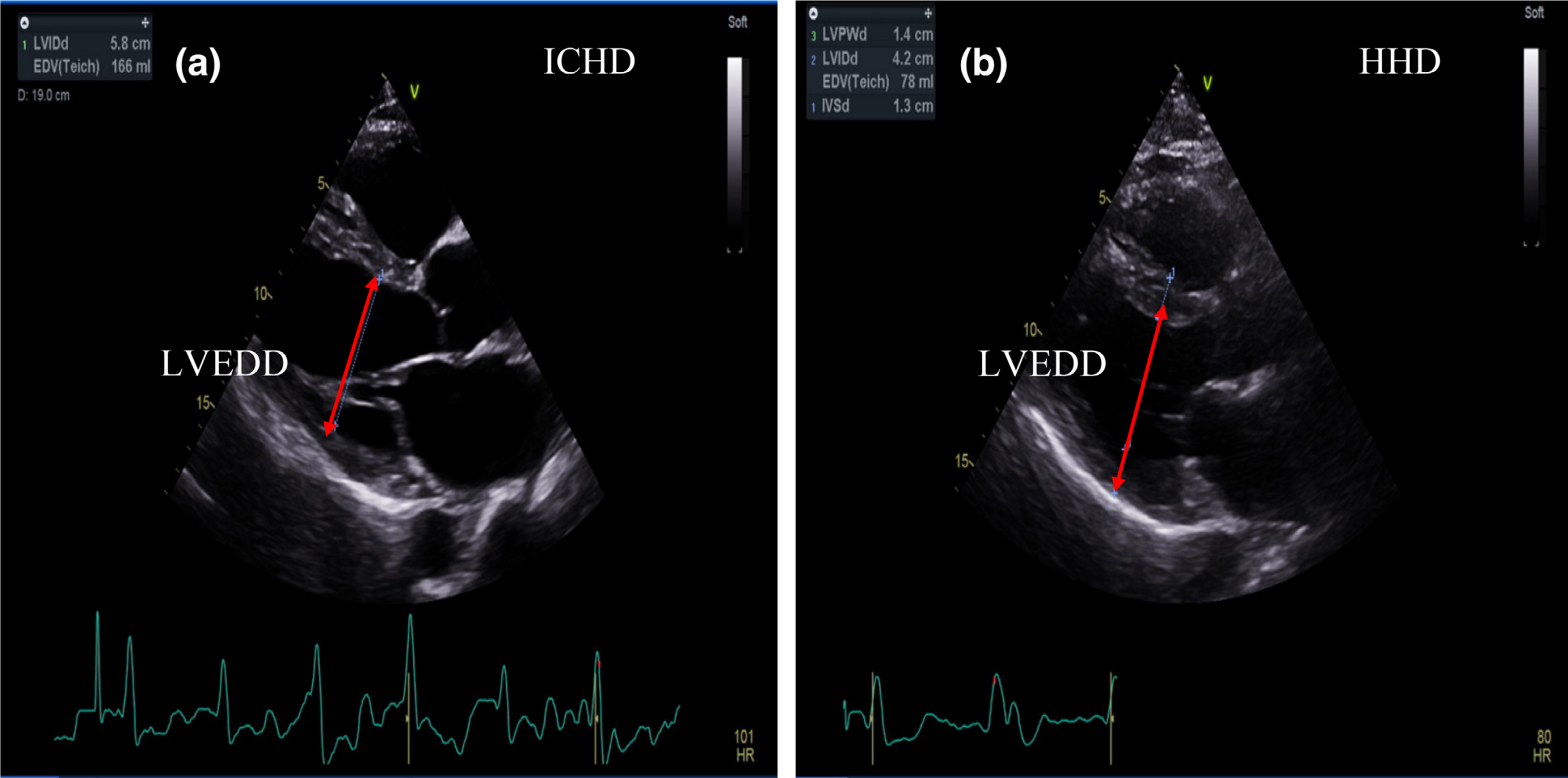
Linear measurements of left ventricular (LV) dimensions in systole and diastole, assessed through the use of transthoracic echocardiography in an adult with end‐stage kidney disease (ESKD) who was receiving home hemodialysis (HHD) (Plot a) and an adult with ESKD who was receiving in‐centre hemodialysis (ICHD) (Plot s). A significantly larger (*p* = 0.03) LV end diastolic diameter can be seen in the adult receiving HHD.

Resting parameters of cardiac structure and function from transthoracic echocardiography are presented in Table 2. In the total group, LVEF was lower (in 7/17 participants) and *Q̇* (in 9/17 participants) and LV_mass_ (in 11/17 participants) were increased when compared to British Society of Echocardiography normative reference values for healthy adults (Echocradiography BS of. Protocols and guidelines, 2021). Resting left ventricular end‐diastolic diameter (LVEDD; Figure 1) was significantly greater (*p* = 0.03, *ES* = 0.53) in adults receiving HHD compared to those receiving ICHD. No significant between groups differences were evident in *Q̇* (*p* = 0.15, *ES* = 0.43) or SV (*p* = 0.41, ES = 0.25) at rest between the HHD and ICHD subgroups. Both serum [NTproBNP] and [TnI] were outside the normal ranges (Corteville et al., 2007) post‐hemodialysis in adults with ESKD, however no differences were present between adults receiving HHD or ICHD (*p* = 0.70 and 0.82 for [NTproBNP] and [TnI], respectively).

**TABLE 2:** Cardiac structure and function in adults with end‐stage kidney disease (ESKD) receiving either home‐ or in‐centre hemodialysis.

	End‐stage kidney disease		Treatment			
	Total group (*n* outside of normative range)	Norm. Values^a^	ICHD	HHD	*p‐*value	ES
Echocardiography						
LVEF (%)	52.5 ± 11.9 (7)	≥ 55	51.3 ± 6.7	52.9 ± 13.4	0.35	0.23
SV (mL)	74.0 ± 20.4 (4)	50–100	76.6 ± 15.4	72.8 ± 22.9	0.69	0.10
*Q̇* (L·min^−1^·BSA^−1^)	3.9 ± 1.1 (9)	5–6	3.6 ± 0.9	4.0 ± 1.3	0.94	0.02
LV mass (g)	233.3 ± 80.0 (9)	72–219	222.4 ± 29.8	236.9 ± 91.8	0.90	0.03
LV mass/BSA (g·m^2^)	124.1 ± 43.3 (11)	40–110	109.6 ± 18.2	128.9 ± 48.7	0.33	0.24
LVEDD (mm)	41.6 ± 10.2 (1)	37–56	30.5 ± 13.7	45.3 ± 5.6	0.03*	0.53*
LVESD (mm)	33.8 ± 7.0 (3)	22–41	31.4 ± 6.4	34.8 ± 7.3	0.24	0.28
LV systolic volume (mL)	60.1 ± 44.4 (3)	15–62	45.0 ± 8.7	65.1 ± 50.7	0.31	0.25
LV diastolic volume (mL)	122.0 ± 51.9 (2)	53–156	100.3 ± 23.5	129.2 ± 57.7	0.31	0.25
Mild LVH (*n*)	11	‐	4	7	‐	‐
Moderate LVH (*n*)	2	‐	1	1	‐	‐
Severe LVH (*n*)	1	‐	0	1	‐	‐
GLS (%)	−13.2 ± 4.6 (0)	< 21%	−14.9 ± 0.9	−12.3 ± 5.6	0.40	0.20
LA volume/BSA (mL·m^−2^)	28.1 ± 15.6 (2)	< 34	55.8 ± 6.9	54.3 ± 39.2	0.33	0.24
TAPSE (cm)	1.9 ± 0.5 (9)	≥ 1.7	2.14 ± 0.5	1.9 ± 0.5	0.17	0.33
Cardiac biomarkers (post‐HD)						
Troponin I (ng·L^−1^)	16.4 ± 11.7 (6)	< 16.0	14.4 ± 7.0	18.4 ± 13.2	0.82	0.06
NTproBNP (pg·mL^−1^)	6908 ± 8698 (14)	< 125	4926 ± 2759	8360 ± 8965	0.70	0.10
B2M (mg·L^−1^)	9.9 ± 5.3 (3)	7.0–18.0	23.6 ± 8.7	19.3 ± 6.7	0.59	0.15

Abbreviations: B2M, beta‐2 Microglobulin; EF, ejection fraction; ES, effect size; GLS, global longitudinal strain; HHD, home hemodialysis; ICHD, in‐centre hemodialysis; LA, left atrium; LV, left ventricle; LVEDD, left ventricular end‐diastolic diameter; LVESD, left ventricular end systolic diameter; LVH, left ventricular hypertrophy; NTproBNP, B‐type natriuretic peptide; *Q̇*, cardiac output; SV, stroke volume; TAPSE, tricuspid annular plane systolic excursion.

^a^ Normative biochemistry values for healthy adults obtained from Corteville et al. (2007), Normative echocardiography values obtained from the British Society of Echocardiography. Harkness et al. (2020).

* Denotes statistical significance between the HHD and ICHD subgroups.

### 3.2 Exercise testing

#### 3.2.1 CPET

Resting and exercise parameters from submaximal cycling CPET are presented in Tables 3 and 4. Of the 17 participants, 11 (*n* = 3 ICHD) completed CPET; six were considered medically contraindicated for exercise testing. V̇O_2_ at the GET was 0.88 ± 0.22 L·min^−1^, equivalent to 39 ± 8% predicted V̇O_2peak_. The mean V̇O_2_ at end‐exercise (RPE 15) was 1.07 ± 0.3 L·min^−1^, equivalent to 53 ± 11% predicted V̇O_2peak_. The mean ramp duration in the total group of dialysing adults was 635 ± 187 seconds. Resting *V̇*CO_2_ was significantly higher in the ICHD subgroup (0.48 ± 0.09 L·min^−1^) compared to HHD (0.34 ± 0.09 L·min^−1^, *p* = 0.02, ES = 0.68). All participants terminated exercise upon reaching an RPE of 15 on the Borg scale (Borg, 1982).

**TABLE 3:** Cardiopulmonary responses at rest and during unloaded cycling exercise.

Variable	Total group	ICHD (*n* = 3)	HHD (*n* = 8)	*p‐*value	ES
Rest					
V̇O_2_ (L·min^−1^)	0.46 ± 0.14	0.57 ± 0.14	0.41 ± 0.12	0.15	0.43
*V̇*CO_2_ (L·min^−1^)	0.38 ± 0.11	0.48 ± 0.09	0.34 ± 0.09	0.02*	0.68*
RER	0.84 ± 0.12	0.87 ± 0.14	0.82 ± 0.11	0.68	0.12
*F*_R_ (breaths·min^−1^)	17 ± 2	17 ± 2	17 ± 2	1.00	0.00
O_2_ pulse (mL·min^−1^·beat^−1^)	5.7 ± 1.7	7.6 ± 0.5	4.9 ± 1.4	0.05^+^	0.58*
SV (mL·beat^−1^)	87.7 ± 18.7	97.1 ± 19.9	84.2 ± 18.3	0.41	0.25
*Q̇* (L·min^−1^)	6.8 ± 0.9	7.1 ± 0.4	6.6 ± 1.1	0.15	0.43
HR (beats·min^−1^)	79 ± 12	74 ± 19	81 ± 10	0.41	0.25
SBP (mmHg)	117 ± 15	116 ± 20	117 ± 15	0.92	0.03
DBP (mmHg)	78 ± 12	72 ± 13	81 ± 12	0.36	0.28
Unloaded cycling (10 watts)					
V̇O_2_ (L·min^−1^)	0.55 ± 0.15	0.70 ± 0.19	0.5 ± 0.1	0.15	0.43
*V̇*CO_2_ (L·min^−1^)	0.47 ± 0.13	0.59 ± 0.18	0.42 ± 0.08	0.18	0.40
RPE	7 ± 1	8 ± 1	7 ± 1	0.07^+^	0.54
RER	0.84 ± 0.08	0.84 ± 0.05	0.85 ± 0.09	0.68	0.12
*V̇*_E_ (L·min^−1^)	18.1 ± 5.7	22.6 ± 6.9	16.4 ± 4.5	0.15	0.43
*F*_R_ (breaths·min^−1^)	18 ± 2	18 ± 4	18 ± 2	0.84	0.06
O_2_ pulse (mL·min^−1^·beat^−1^)	7.1 ± 2.71	9.7 ± 3.6	5.9 ± 1.3	0.05^+^	0.58*
SV (mL·beat^−1^)	87.6 ± 17.8	97.8 ± 22.4	83.8 ± 15.2	0.41	0.25
*Q̇* (L·min^−1^)	6.7 ± 0.9	7.2 ± 1.2	6.5 ± 0.7	0.41	0.24
HR (beats·min^−1^)	82 ± 13	75 ± 20	84 ± 9.26	0.54	0.18
SBP (mmHg)	125 ± 15	131 ± 9	123 ± 17	0.84	0.06
DBP (mmHg)	80 ± 10	80 ± 6	79 ± 11	0.79	0.08

Abbreviations: DBP, diastolic blood pressure; ES, effect size; *f*_R_, breathing frequency; HHD, home hemodialysis; HR, heart rate; ICHD, in‐centre hemodialysis; *Q̇*, cardiac output; RER, respiratory exchange ratio; RPE, subjective rating of perceived exertion; SBP, systolic blood pressure; SV, stroke volume; *V̇*CO_2_, rate of carbon dioxide production; *V̇*_E_, minute ventilation; V̇O_2_, rate of oxygen consumption.

* Denotes statistical significance between HHD and ICHD.

^+^ Denotes a trend toward statistical significance between HHD and ICHD.

**TABLE 4:** Submaximal exercise responses at the gas exchange threshold and end‐exercise (RPE of 15 on the Borg scale).

CPET variable	Total group	ICHD (*n* = 3)	HHD (*n* = 8)	*p‐*value	ES
Gas exchange threshold					
V̇O_2_ (L·min^−1^)	0.88 ± 0.22	0.95 ± 0.34	0.93 ± 0.18	0.84	0.06
V̇O_2_ (% predicted)	39 ± 8	39 ± 8	38 ± 10	0.88	0.02
V̇O_2_ (mL·min^−1^˙ kg^−1^)	11.41 ± 3.02	10.29 ± 2.62	11.83 ± 3.21	0.61	0.14
V̇CO_2_ (L.min^−1^)	0.74 ± 0.15	0.79 ± 0.19	0.79 ± 0.15	0.92	0.03
WR (W)	50 ± 17	54 ± 22	52 ± 17	0.84	0.06
RER	0.80 ± 0.08	0.87 ± 0.13	0.86 ± 0.06	0.91	0.03
*V̇*_E_ (L·min^−1^)	26.7 ± 7.0	29.2 ± 7.6	27.9 ± 7.3	0.68	0.12
*F*_R_ (breaths·min^−1^)	22 ± 9	33 ± 15	20 ± 5	0.22	0.37
*V̇*_E_/*V̇*CO_2_ slope	28.18 ± 10.23	21.93 ± 4.09	30.54 ± 11.03	0.09	0.48
O_2_ pulse (mL·min^−1^·beat^−1^)	8.7 ± 1.8	10.7 ± 1.6	8.8 ± 1.7	0.15	0.43
SpO_2_ (%)	97 ± 1	96 ± 1	97 ± 1	0.92	0.07
SV (mL·beat^−1^)	117.7 ± 22.1	141.4 ± 13.8	108.7 ± 17.6	0.01*	0.74*
*Q̇* (L·min^−1^)	11.2 ± 2.4	12.6 ± 3.7	10.6 ± 1.8	0.31	0.31
HR (beats·min^−1^)	89 ± 17	86 ± 23	99 ± 15	0.31	0.31
SBP (mmHg)	129 ± 27	118.0 ± 24.5	145.50 ± 25.17	0.15	0.43
DBP (mmHg)	80 ± 14	75.67 ± 14.74	89.38 ± 12.18	0.31	0.31
End exercise					
V̇O_2_ (L·min^−1^)	1.07 ± 0.3	1.23 ± 0.47	1.01 ± 0.23	0.36	0.28
V̇O_2_ (% predicted)	53 ± 11	54 ± 12	53 ± 12	0.91	0.01
V̇O_2_ (mL·min^−1^˙·kg^−1^)	13.81 ± 4.56	15.35 ± 5.30	13.23 ± 4.50	0.31	0.30
*V̇*CO_2_ (L·min^−1^)	1.08 ± 0.29	1.27 ± 0.4	1.00 ± 0.23	0.18	0.40
WR (W)	80 ± 30	95 ± 40	75 ± 26	0.36	0.28
Δ*V*O_2_/ΔWR (mL·min^−1^·W^−1^)	7.15 ± 3.77	4.88 ± 5.46	8.01 ± 2.94	0.24	0.40
Exercise duration (s)	635 ± 187	642 ± 209	616 ± 145	0.27	0.34
RER	1.01 ± 0.05	1.05 ± 0.07	1.00 ± 0.05	0.26	0.38
*V̇*_E_ (L·min^−1^)	37.7 ± 7.6	41.2 ± 10.1	36.6 ± 6.9	0.41	0.25
*F*_R_ (breaths·min^−1^)	25 ± 6	24 ± 1	25 ± 7	0.84	0.06
HR (beats·min^−1^)	112 ± 20	108 ± 27	114 ± 19	0.76	0.09
O_2_ pulse (mL·min^−1^·beat^−1^)	10.8 ± 2.6	13.3 ± 2.6	9.8 ± 1.9	0.05^+^	0.58*
SV (mL·beat^−1^)	124.3 ± 27.9	156.8 ± 23.5	112.1 ± 18.4	0.01*	0.74*
*Q̇* (L·min^−1^)	12.6 ± 3.1	14.4 ± 5.0	11.9 ± 2.1	0.41	0.25
SBP (mmHg)	146 ± 24	142 ± 1	146 ± 25	0.15	0.43
DBP (mmHg)	88 ± 12	81 ± 1.5	89 ± 12	0.79	0.08

Abbreviations: DBP, diastolic blood pressure; ES, effect size; *f*_R_, breathing frequency; HHD, home hemodialysis; HR, heart rate; ICHD, in‐centre hemodialysis; *Q̇*, cardiac output normalized to body surface area (BSA), CI; RER, respiratory exchange ratio; RPE, subjective rating of perceived exertion; SBP, systolic blood pressure; SpO_2_, arterial O_2_ saturation; SV, stroke volume normalized to BSA, SVI; *V̇*CO_2_, rate of carbon dioxide production; *V̇*_E_, minute ventilation; V̇O_2_, rate of pulmonary oxygen uptake; WR, work rate.

* Denotes statistical significance (*p* < 0.05) between the HHD and ICHD subgroups.

^+^ Denotes a trend toward statistical significance (*p* < 0.07) between HHD and ICHD.

Non‐invasively measured SV was significantly lower in adults receiving HHD (141.4 ± 13.8 mL·beat^−1^) versus ICHD (108.7 ± 17.6 mL·beat^−1^, *p* = 0.01, ES = 0.74) at the GET and end‐exercise (156.8 ± 23.5 mL·beat^−1^ vs. 112.1 ± 18.4 mL·beat^−1^, *p* = 0.01, ES = 0.74). A large ES was evident for the difference between ICHD and HHD for estimated SV (O_2_ pulse) during unloaded cycling (*p* = 0.05, ES = 0.58) and end‐exercise (*p* = 0.05, ES = 0.58).

#### 3.2.2 Functional tests

Functional test parameters, relative to appropriate normative references values (Amirjani et al., 2007; Ramírez‐Vélez et al., 2020; Strassmann et al., 2013; Wang et al., 2018), are in Table 5. Of 17 participants, 16 (*n* = 5 ICHD) completed all functional field tests, with all achieving scores below normative reference values for the STS5 and 1‐minute STS. No significant differences were evident between the HHD and ICHD subgroups for any functional test outcome.

**TABLE 5:** Functional exercise capacity of adults receiving ICHD or HHD compared to normative values.

	Normative reference values^a^	Total group (*n* outside of normative range)	ICHD (*n* = 5)	HHD (*n* = 11)	*p*‐value	ES
SPPB						
STS 5 (s)	1.1–3.3	9.7 ± 4.9 (16)	6.3 ± 7.0	11.3 ± 2.9	0.13	0.41
Balance test (points)	2.8–4.4	3.4 ± 1.3 (3)	2.6 ± 1.9	3.8 ± 0.6	0.11	0.42
Gait speed test (s)	1.4–2.4	1.82 ± 0.65 (3)	1.63 ± 0.96	1.92 ± 0.49	0.96	0.02
Total (points)	7.2–11.2	10 ± 3 (7)	8 ± 5	11 ± 2	0.25	0.31
Sit‐to‐stand 5 power (W·kg^−1^) Moberg's picking up test		3.1 ± 1.6	4.2 ± 3.4	2.8 ± 0.6	0.18	0.37
Eyes open (s)	15.4–18.0	13.7 ± 6.6 (2)	17.8 ± 11.1	11.8 ± 2.5	0.69	0.11
Eyes closed (s)	27.8–31.5	35.6 ± 30.7 (5)	49.97 ± 52.65	29.14 ± 12.43	0.96	0.02
Handgrip strength (kg)	35–45	33.1 ± 7.9 (8)	31.8 ± 10.5	36.2 ± 6.0	0.50	0.13
1‐minute STS (reps)	61–63	22 ± 14 (16)	17 ± 17	25 ± 12	0.49	0.13

Abbreviations: ES, effect size; HHD, home hemodialysis; ICHD, in‐centre hemodialysis; reps, repetitions; SPPB, short physical performance battery; STS, sit‐to‐stand.

^a^ Normative reference values obtained from Ramirez‐Velez et al. (2020), Amirjani et al. (2007), Wang et al. (2018), and Strassman et al. (2013).

### 3.3 Muscle oxygenation

Peripheral muscle oxygenation parameters, at rest and at the GET, are in Table 6. No significant differences were found in any markers of peripheral muscle oxygenation between groups or between baseline and GET.

**TABLE 6:** Peripheral muscle oxygenation of the *m. vastus lateralis* at baseline and gas exchange threshold in adults receiving ICHD or HHD.

CPET variable	Total group	ICHD (*n* = 3)	HHD (*n* = 8)	*p* value	ES
Baseline					
[O_2_Hb] (a.u.)	10.11 ± 14.73	0.92 ± 3.61	13.56 ± 16.02	0.11	0.32
[HHb] (a.u.)	9.68 ± 15.54	0.14 ± 3.59	13.26 ± 16.97	0.11	0.32
[tHb] (a.u.)	19.79 ± 29.91	1.06 ± 1.64	26.81 ± 32.72	0.07	0.46
TSI (%)	75.99 ± 12.41	88.13 ± 14.39	71.44 ± 6.59	0.17	0.46
V̇O_2_ (L·min^−1^)	0.52 ± 0.08	0.56 ± 0.12	0.50 ± 0.07	0.51	0.15
WR (W)	0.30 ± 0.38	0.78 ± 0.39	0.13 ± 0.17	0.09	0.43
Gas exchange threshold					
[O_2_Hb] (a.u.)	11.17 ± 15.94	3.04 ± 6.87	14.21 ± 17.49	0.16	0.36
[HHb] (a.u.)	9.46 ± 15.89	1.09 ± 3.80	12.61 ± 17.75	0.12	0.29
[tHb] (a.u.)	20.63 ± 31.31	4.12 ± 6.77	26.82 ± 35.03	0.20	0.35
TSI (%)	77.43 ± 13.56	88.44 ± 14.21	73.29 ± 11.56	0.12	0.29
V̇O_2_ (L·min^−1^)	0.80 ± 0.20	0.87 ± 0.23	0.78 ± 0.20	0.56	0.13
WR (W)	42.09 ± 16.24	41.67 ± 19.66	42.25 ± 16.32	0.97	0.04
%HHb/%V̇O_2_	4.75 ± 5.88	4.23 ± 10.67	4.95 ± 4.08	0.92	0.08

Abbreviations: a.u., arbitrary units; HHb, deoxy‐hemoglobin; L, Liters; O_2_Hb, oxy‐hemoglobin, tHb, total hemoglobin, TSI, tissue saturation index; V̇O_2_, rate of oxygen consumption; WR, work rate.

### 3.4 Mechanistic associations between cardiac structure and function and measures of physical fitness

**FIGURE 2:**
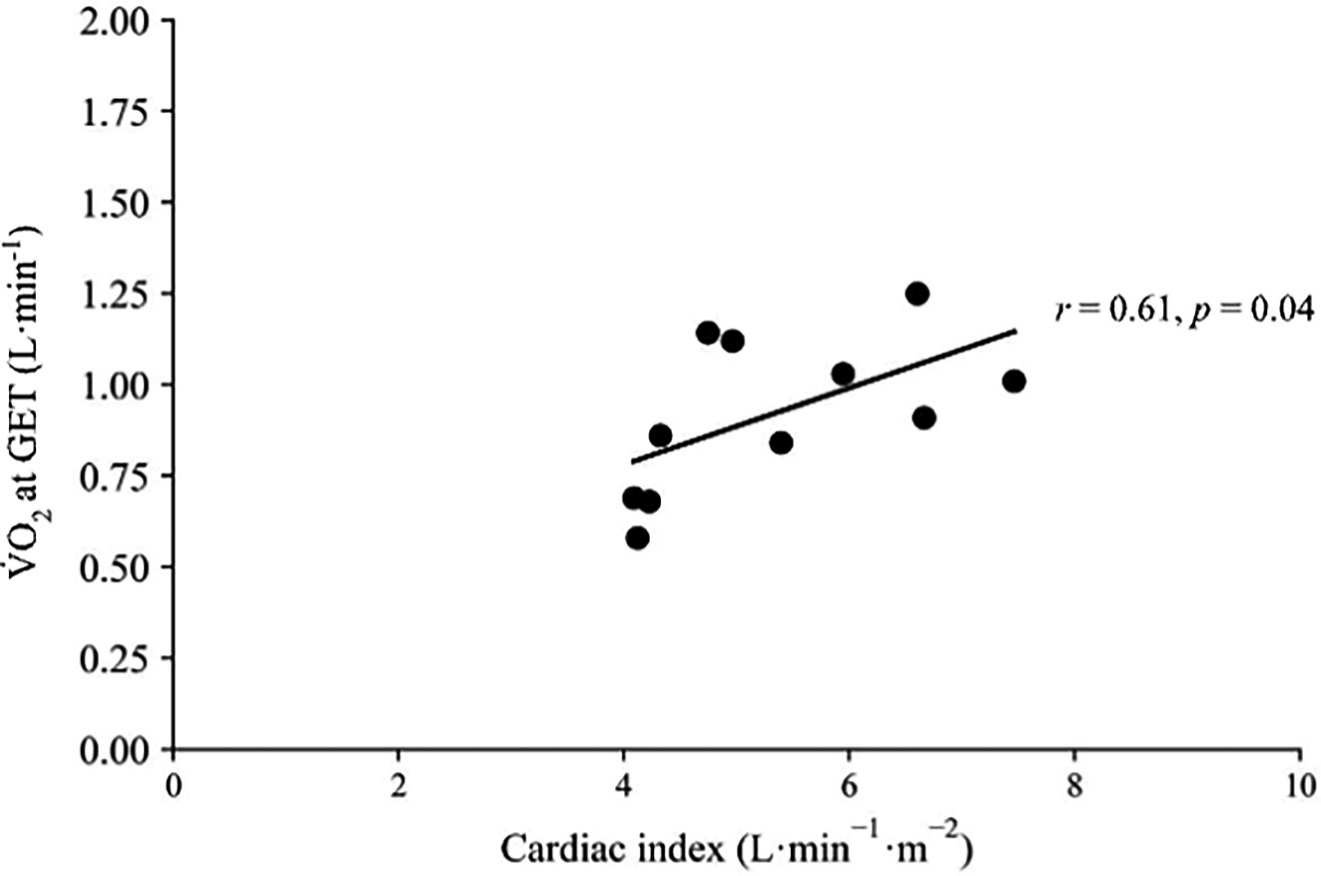
Mechanistic association between cardiac index and pulmonary oxygen uptake (V̇O_2_) at the gas exchange threshold in chronically dialysing adults (in‐centre and home‐hemodialysis) living with end‐stage kidney disease (ESKD).

**FIGURE 3:**
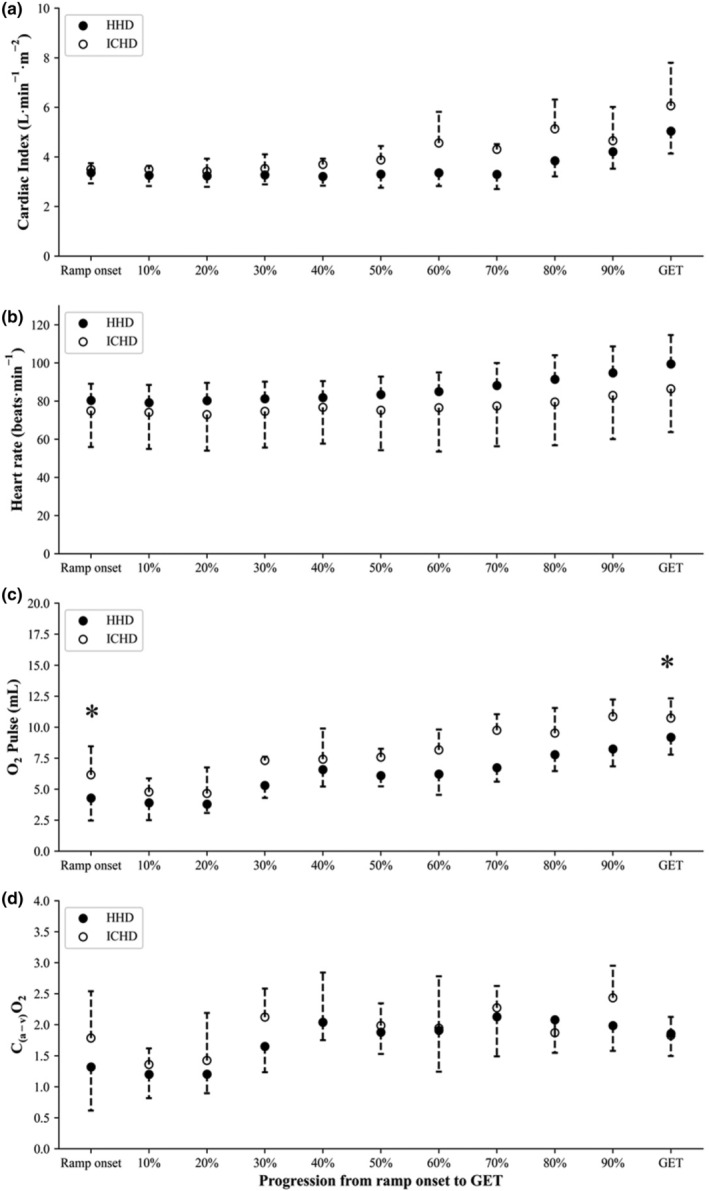
(a) Cardiac index in adults receiving in‐centre hemodialysis (ICHD) or home hemodialysis (HHD) from baseline to the gas exchange threshold (GET) during submaximal ramp incremental cycling exercise; (b) heart rate response of adults receiving ICHD or HHD from baseline to the GET during ramp incremental cycling; (c) O_2_ pulse response of adults receiving ICHD or HHD from baseline to the GET during ramp incremental cycling exercise; (d) normalized arteriovenous O_2_ content difference [*C*_(a‐v)_O_2_] during ramp incremental cycling exercise to the GET. *p < 0.05, significant mean difference between adults receiving ICHD or HHD.

Within the total cohort, a statistically significant, moderate, positive association between the *Q̇* and relative V̇O_2_ (*r* = 0.61, *p* = 0.04; Figure 2) at the GET, was found, with associations trending toward significance between resting LV mass index and V_E_ at the GET, and handgrip strength (Table 7). No other significant associations existed between aerobic fitness and mechanistically important variables of cardiac structure and function, nor mechanistic associations between body composition and peripheral muscular strength (Figure 3).

**TABLE 7:** Correlations between fitness and mechanistic outcomes in adults with kidney failure receiving either HHD or ICHD.

Variables	Correlation (*r*)	*p* value
Resting LV mass/BSA versus V̇O_2_ at GET	−0.38	0.25
Resting LV mass/BSA versus *V́*_E_ at GET	−0.58	0.06^+^
Resting LV mass/BSA versus WR at GET	0.31	0.37
Resting LV mass/BSA versus handgrip strength	−0.54	0.08^+^
Cardiac index at GET versus V̇O_2_ at GET	0.61	0.04*
Resting EF versus V̇O_2_ at GET	0.55	0.10
Resting EF versus CI at GET	0.03	0.93
Resting LVEDD versus V̇O_2_ at GET	0.03	0.93

* Statistical significance at the *p* ≤ 0.05 level.

^+^ Statistical trend toward significance (i.e., *p* = 0.05–0.1).

## 4 DISCUSSION

This study uniquely combined laboratory and field exercise tests with echocardiography, cardiac biomarkers, thoracic bioreactance, and NIRS to investigate central and peripheral mechanisms of exercise (dys)function in adults with ESKD receiving in‐centre and home‐based dialysis. Key findings revealed impaired ventricular function and LVH in adults with ESKD, which were mechanistically linked to the severely reduced exercise capacity. Additionally, adults with ESKD showed an inability to adequately raise tissue oxygenation and O_2_ utilization during exercise. Both aerobic capacity and muscular function were below normative values in this population, with similar dysfunction observed in both ICHD and HHD patients.

LVH, commonly reported in ESKD, results partly from increased cardiac afterload and pulse wave velocity, associated with the increased *Q̇* and BP typically characterizing adults with ESKD (Jankowski et al., 2021). Echocardiography in the present study identified reduced EF, *Q̇* and evidence of LVH compared to established norms. This is important, given known negative associations between cardiac parameters and adverse cardiovascular outcomes (LVEF is associated with increased CV events [hazard ratio 2.01]) in adults with ESKD (Burton et al., 2009). These abnormalities were present in most participants, regardless of dialysis type, although LVEDD was significantly larger in adults receiving HHD, suggesting ventricular insufficiency in ICHD patients (Aleong et al., 2015). Further to this, a trend toward a significant association between resting LV mass index and V_E_ at the GET, as well as resting LV mass index and handgrip strength support a mechanistic link between cardiac dysfunction and compromised physical fitness in this population. These findings were contrary to previous evidence suggesting improvements in cardiac structure and function, in particular LVH and LV mass index, following a switch from conventional to more frequent hemodialysis (Susantitaphong et al., 2012). Abnormal [Troponin] and [NTproBNP] further characterized abnormal cardiac function in the present study cohort, highlighting the need for prospective evaluations comparing ICHD and HHD over time.

Using CPET to characterize physiological (dys) function in kidney disease has been relatively scarce. This study used non‐invasive thoracic bio‐reactance during CPET, revealing poor cardiac function that is mechanically linked to reduced exercising aerobic capacity. CPET was terminated prior to exhaustion, due to cardiac risk, however the reduced GET (39% predicted V̇O_2peak_) (Wasserman & Whipp, 1975), still supports previous findings of exercise limitations in ESKD (Ting et al., 2015) and was still reduced compared to non‐matched reference values of between 50% and 60% (Koch et al., 2009). Given that both peak and submaximal aerobic fitness appear to be associated with mortality in this population (Ting et al., 2014), the reduced cardiorespiratory fitness reported herein is of clinical importance. CPET was terminated before exhaustion due to cardiac risk, but the reduced GET (39% predicted V̇O_2_) still supports previous findings of exercise limitations in ESKD.

The present study also supports earlier reports of chronotropic incompetence in adults with ESKD during exercise (McGuire et al., 2020), with a mean HR of 89 ± 17 beats·min^−1^ at the GET. In a large sample of healthy individuals (Agostoni et al., 2017), a peak *Q̇* comparable to our findings at GET (13.2 ± 3.5 L·min^−1^ vs. 11.16 ± 2.44 L·min^−1^ respectively) was achieved at a V̇O_2_ of 95% predicted, compared to our 39%. Our findings support a cardiovascular limitation during exercise in individuals with ESKD, with lower SV and *Q̇*, hindering O_2_ delivery to the muscles, in line with prior evidence (McGuire et al., 2020). Adults receiving HHD had greater LVEDD compared to those on ICHD, however ICHD recipients exhibited significantly higher SV at both the GET and end‐exercise, indicating increased chronotropic incompetence in the ICHD group.

Exercise capacity is not only determined by the ability to uptake O_2_ centrally, but also to extract and utilize locally within the muscle. Reduced diffusion of O_2_ from the capillary to mitochondria (Stray‐Gundersen et al., 2016), and a diminished ability to increase peripheral muscle oxygenation during submaximal exercise (MacDonald et al., 2012), have previously been reported in ESKD. In the present study, NIRS indicated a blunted TSI% response, suggesting impaired muscle oxygenation and O_2_ utilization during exercise. This supports previous research suggesting an impaired microvascular hyperaemic response in adults with kidney disease (Theodorakopoulou et al., 2023). The lack of any significant differences in TSI between baseline and GET in this study suggest a reduced ability to increase O_2_ utilization in response to exercise. In addition to this, a recorded V̇O_2_ equal to 53% predicted V̇O_2peak_ within this study, at 15/20 on the Borg scale suggests that whilst individuals may have been able to continue exercising, impaired O_2_ extraction within the working muscles led to increased lactate production, supported by a RER of 1.01 ± 0.05 at test termination. Previously, Wilkinson et al. (2019), used NIRS to explore skeletal muscle O_2_ saturation in individuals with CKD finding that, compared to healthy controls, adults with CKD demonstrated a greater deoxygenation rate, fatiguing sooner than controls, and taking significantly longer (83.1 ± 13.5 seconds vs. 36.8 ± 33.0 seconds, respectively, *p* = 0.01) to recover from their exercise. Findings from this study, and previous research suggest that adults with CKD, in particular ESKD, have a reduced ability to adequately raise TSI and O_2_ utilization in response to exercise, which may further contribute to exercise tolerance.

Functional exercise tests, such as the SPPB, 1‐minute STS, gait speed, and handgrip dynamometry, offer complementary insights into fitness and ADL capability (Arikan et al., 2015; López‐Montes et al., 2020). Adults receiving ICHD in the present study showed abnormal SPPB scores, indicating greater risk of physical deterioration. Typically, an abnormal SPPB score is defined as ≤9 points (Almugbel et al., 2021), therefore the mean score in this study for HHD is normal. However, the mean of the ICHD sub‐group was abnormal, demonstrating greater risk of deteriorating physical ability (Vasunilashorn et al., 2009). Moreover, both HHD and ICHD patients had lower handgrip strength and STS results, reflecting reduced aerobic capacity and muscular strength, consistent with previous findings in individuals with CKD (Vogt et al., 2016). Findings from this study suggest limited differences between HHD and ICHD, although caution is warranted given the moderate effects found in the balance test and STS5, respectively. Importantly, these impairments to functional ability have the potential to increase falls risk, reduce independence, quality of life and impair activities of daily living.

The findings of the present study must be considered in the context of a number of methodological limitations. First, although some significant differences and large effects were found and the individuals receiving ICHD and HHD were closely matched, when accounting for the excess fluid carried by those receiving ICHD, the small, relatively heterogeneous and unbalanced groups limit the ability to generalize the results and does not allow for firm inter‐group conclusions to be made. Therefore, larger studies utilizing multimodal exercising testing are needed to confirm findings from this study, in particular focusing on defining the minimum clinically important difference for these tests between different dialysis modalities. Second, this study did not recruit and compare to any matched controls in characteristics which are known to influence physical performance, such as age or sex, and the low sample size prohibited the comparison with normative reference values and therefore future research needs to compare to matched controls to confirm these findings. Finally, whilst this study demonstrates the value of a number of non‐invasive techniques, the low sample size and gender imbalance present within this study limits the generalisability of the findings and their applicability to the wider ESKD population, as well as potentially introducing bias which may affect the interpretability of the results. We performed a post‐hoc power calculation based upon the differences in LVEDD. For 90% power and an *α*‐level set at *p* = 0.05 (two tailed), with an effect size of 0.53, 36 individuals would be needed. Future trials should therefore aim to recruit at least 40 participants to account for loss to follow‐up.

In conclusion, this study used multimodal exercise testing to examine impairments in aerobic fitness, peripheral muscle function, and fine motor ability in adults with ESKD, with limited differences found between those receiving ICHD vs. HHD. Submaximal fitness (GET) was reduced, with cardiac limitations caused by ventricular insufficiency and cardiac damage, and impaired O_2_ utilization at the muscle, contributing to multi‐factorial exercise dysfunction in adults with ESKD. These findings provide novel insights into the challenges faced by adults with ESKD.

## FUNDING INFORMATION

This study was funded by NxStage Medical Inc.

## ETHICS STATEMENT

Ethics approval was provided by the South Central – Oxford B Research Ethics Committee (REC reference: (Chan et al., 2019)/SC/0684) and Health Research Authority and the study was pre‐registered on ClinicalTrials.gov (NCT03925454).

## Data Availability

The data that support the findings of this study are available on reasonable request from the corresponding author.

## References

[phy270050-bib-0001] Agostoni, P. , Vignati, C. , Gentile, P. , Boiti, C. , Farina, S. , Salvioni, E. , et al. (2017). Reference values for peak exercise cardiac output in healthy individuals. Chest, 151(6), 1329–1337. 28108178 10.1016/j.chest.2017.01.009

[phy270050-bib-0002] Aleong, R. G. , Mulvahill, M. J. , Halder, I. , Carlson, N. E. , Singh, M. , Bloom, H. L. , Dudley, S. C. , Ellinor, P. T. , Shalaby, A. , Weiss, R. , Gutmann, R. , Sauer, W. H. , Narayanan, K. , Chugh, S. S. , Saba, S. , & London, B. (2015). Left ventricular dilatation increases the risk of ventricular arrhythmias in patients with reduced systolic function. Journal of the American Heart Association, 4(8), e001566. 26231842 10.1161/JAHA.114.001566 PMC4599449

[phy270050-bib-0003] Almugbel, F. A. , Timilshina, N. , Papadopoulos, E. , Al‐Showbaki, L. , & Alibhai, S. M. H. (2021). The role of grip strength and short physical performance battery test in predicting chemotherapy‐related outcomes in older adults with cancer. Journal of geriatric oncology, 31, S1054. 10.1016/j.jgo.2021.12.002 34924306

[phy270050-bib-0004] Amirjani, N. , Ashworth, N. L. , Gordon, T. , Edwards, D. C. , & Chan, K. M. (2007). Normative values and the effects of age, gender, and handedness on the Moberg pick‐up test. Muscle & Nerve, 35(6), 788–792. 17326120 10.1002/mus.20750

[phy270050-bib-0005] Arikan, H. , Yatar, İ. , Calik‐Kutukcu, E. , Aribas, Z. , Saglam, M. , Vardar‐Yagli, N. , Savci, S. , Inal‐Ince, D. , Ozcelik, U. , & Kiper, N. (2015). A comparison of respiratory and peripheral muscle strength, functional exercise capacity, activities of daily living and physical fitness in patients with cystic fibrosis and healthy subjects. Research in Developmental Disabilities, 45–46, 147–156. 10.1016/j.ridd.2015.07.020 26241869

[phy270050-bib-0006] Bayoumi, M. M. , & Alwakeel, J. (2015). Impacts of exercise programs on hemodialysis Patients' quality of life and physical fitness. Quality in Primary Care, 23(4), 192–200.

[phy270050-bib-0007] Beaver, W. L. , Wasserman, K. , & Whipp, B. J. (1986). A new method for detecting anaerobic threshold by gas exchange. Journal of Applied Physiology, 60(6), 2020–2027. 3087938 10.1152/jappl.1986.60.6.2020

[phy270050-bib-0008] Borg, G. A. (1982). Psychophysical bases of perceived exertion. Medicine and Science in Sports and Exercise, 14(5), 377–381. 7154893

[phy270050-bib-0009] Burton, J. O. , Jefferies, H. J. , Selby, N. M. , & McIntyre, C. W. (2009). Hemodialysis‐induced cardiac injury: Determinants and associated outcomes. Clinical Journal of the American Society of Nephrology, 4(5), 914–920. 19357245 10.2215/CJN.03900808 PMC2676185

[phy270050-bib-0010] Chan, K. N. , Chen, Y. , Lit, Y. , Massaband, P. , Kiratli, J. , Rabkin, R. , & Myers, J. N. (2019). A randomized controlled trial of exercise to prevent muscle mass and functional loss in elderly hemodialysis patients: Rationale, study design, and baseline sample. Contemporary Clinical Trials Communications, 1(15), 100365. 10.1016/j.conctc.2019.100365 PMC6536673 31193611

[phy270050-bib-0011] Corteville, D. C. M. , Bibbins‐Domingo, K. , Wu, A. H. B. , Ali, S. , Schiller, N. B. , & Whooley, M. A. (2007). N‐terminal pro–B‐type natriuretic peptide as a diagnostic test for ventricular dysfunction in patients with coronary disease: Data from the heart and soul study. Archives of Internal Medicine, 167(5), 483. 17353496 10.1001/archinte.167.5.483 PMC2770346

[phy270050-bib-0012] Cruz‐Jentoft, A. J. , Bahat, G. , Bauer, J. , Boirie, Y. , Bruyère, O. , Cederholm, T. , Cooper, C. , Landi, F. , Rolland, Y. , Sayer, A. A. , Schneider, S. M. , Sieber, C. C. , Topinkova, E. , Vandewoude, M. , Visser, M. , Zamboni, M. , Writing Group for the European Working Group on Sarcopenia in Older People 2 (EWGSOP2) , & The Extended Group for EWGSOP2 (2019) . (2019). Sarcopenia: Revised European consensus on definition and diagnosis. Age and Ageing, 48(1), 16. 30312372 10.1093/ageing/afy169 PMC6322506

[phy270050-bib-0013] Diesel, W. , Noakes, T. D. , Swanepoel, C. , & Lambert, M. (1990). Isokinetic muscle strength predicts maximum exercise tolerance in renal patients on chronic hemodialysis. American Journal of Kidney Diseases, 16(2), 109–114. 2382645 10.1016/s0272-6386(12)80563-4

[phy270050-bib-0014] Du Bois, D. , & Du Bois, E. F. (1916). Clinical calorimetry: Tenth paper a formula to estimate the approximate surface area if height and weight be known. Nutrition, XVII(6_2), 863–871. 2520314

[phy270050-bib-0015] Echocradiography BS of. Protocols and guidelines . 2021. Retrieved March 2, 2022, from https://www.bsecho.org/Public/Education/Protocols‐and‐guidelines/Public/Education/Protocols‐and‐guidelines.aspx

[phy270050-bib-0016] Garcia, R. S. A. , Lucinda, L. M. F. , Ramos, F. A. , Bueno, G. S. , de Oliveira, G. M. R. , Bonisson, L. S. , Silva, M. A. , Zolli, T. I. , Pinheiro, B. V. , Paula, R. B. , Pazeli, J. M. , & Reboredo, M. M. (2017). Factors associated with functional capacity in hemodialysis patients. Artificial Organs, 41(12), 1121–1126. 10.1111/aor.12938 28568475

[phy270050-bib-0017] Guazzi, M. , Bandera, F. , Ozemek, C. , Systrom, D. , & Arena, R. (2017). Cardiopulmonary exercise testing: What is its value? Journal of the American College of Cardiology, 70(13), 1618–1636. 10.1016/j.jacc.2017.08.012 28935040

[phy270050-bib-0018] Harkness, A. , Ring, L. , Augustine, D. X. , Oxborough, D. , Robinson, S. , & Sharma, V. (2020). Normal reference intervals for cardiac dimensions and function for use in echocardiographic practice: A guideline from the British Society of Echocardiography. Echo Research and Practice, 7(1), 1–18. 32196145 10.1530/ERP-19-0050e PMC8117370

[phy270050-bib-0019] Hopkins, W. G. (2002. Retrieved December 27, 2022, from). New View of Statistics: Effect Magnitudes. https://www.sportsci.org/resource/stats/effectmag.html

[phy270050-bib-0020] Jankowski, J. , Floege, J. , Fliser, D. , Böhm, M. , & Marx, N. (2021). Cardiovascular disease in chronic kidney disease: Pathophysiological insights and therapeutic options. Circulation, 143(11), 1157–1172. 33720773 10.1161/CIRCULATIONAHA.120.050686 PMC7969169

[phy270050-bib-0021] Klaeboe, L. G. , & Edvardsen, T. (2019). Echocardiographic assessment of left ventricular systolic function. Journal of Echocardiography, 17(1), 10–16. 10.1007/s12574-018-0405-5 30390189

[phy270050-bib-0022] Koch, B. , Schäper, C. , Ittermann, T. , Spielhagen, T. , Dörr, M. , Völzke, H. , et al. (2009). Reference values for cardiopulmonary exercise testing in healthy volunteers: The SHIP study. The European Respiratory Journal, 33(2), 389–397. 18768575 10.1183/09031936.00074208

[phy270050-bib-0023] Lankinen, R. , Hakamäki, M. , Metsärinne, K. , Koivuviita, N. , Pärkkä, J. P. , Saarenhovi, M. , Hellman, T. , & Järvisalo, M. J. (2021). Association of maximal stress ergometry performance with troponin T and abdominal aortic calcification score in advanced chronic kidney disease. BMC Nephrology, 22(1), 1–8. 10.1186/s12882-021-02251-y 33541279 PMC7863467

[phy270050-bib-0024] López‐Montes, A. , Martínez‐Villaescusa, M. , Pérez‐Rodríguez, A. , Andrés‐Monpeán, E. , Martínez‐Díaz, M. , Masiá, J. , Giménez‐Bachs, J. M. , & Abizanda, P. (2020). Frailty, physical function and affective status in elderly patients on hemodialysis. Archives of Gerontology and Geriatrics, 1(87), 103976. 10.1016/j.archger.2019.103976 31743824

[phy270050-bib-0025] MacDonald, J. H. , Fearn, L. , Jibani, M. , & Marcora, S. M. (2012). Exertional fatigue in patients with CKD. American Journal of Kidney Diseases, 60(6), 930–939. 22883133 10.1053/j.ajkd.2012.06.021

[phy270050-bib-0026] Matsuo, H. , Dohi, K. , Machida, H. , Takeuchi, H. , Aoki, T. , Nishimura, H. , Yasutomi, M. , Senga, M. , Ichikawa, T. , Kakuta, K. , Mizutani, Y. , Tanoue, A. , Isaka, N. , Oosugi, K. , Koyabu, S. , Sakurai, M. , Fukui, Y. , Kakimoto, H. , Sugimoto, T. , … Ito, M. (2018). Echocardiographic assessment of cardiac structural and functional abnormalities in patients with end‐stage renal disease receiving chronic hemodialysis. Circulation Journal, 82(2), 586–595. 29093429 10.1253/circj.CJ-17-0393

[phy270050-bib-0027] McGuire, S. , Horton, E. J. , Renshaw, D. , Chan, K. , Krishnan, N. , & McGregor, G. (2020). Ventilatory and chronotropic incompetence during incremental and constant load exercise in end‐stage renal disease: A comparative physiology study. American Journal of Physiology. Renal Physiology, 319(3), F515–F522. 10.1152/ajprenal.00258.2020 32744086 PMC7509284

[phy270050-bib-0028] McGuire, S. , Horton, E. J. , Renshaw, D. , Chan, K. , Krishnan, N. , & McGregor, G. (2021a). Cardiopulmonary and metabolic physiology during hemodialysis and inter/ intradialytic exercise. Journal of Applied Physiology, 130(4), 1033–1042. 10.1152/japplphysiol.00888.2020 33507853

[phy270050-bib-0029] Mcguire, S. , Horton, E. J. , Renshaw, D. , Chan, K. , Krishnan, N. , & Mcgregor, G. (2021b. Retrieved Apirl 22, 2021, from). Cardiopulmonary and metabolic physiology during hemodialysis and inter/ intradialytic exercise. http://www.jap.org 10.1152/japplphysiol.00888.2020 33507853

[phy270050-bib-0030] O'Driscoll, J. M. , Edwards, J. J. , Greenough, E. , Smith, E. , May, M. , Gupta, S. , Marciniak, A. , & Sharma, R. (2023). The value of cardiopulmonary exercise testing and stress echocardiography in the prediction of all‐cause mortality in adults with end stage renal disease. European Journal of Sport Science, 23(8), 1800–1809. 10.1080/17461391.2023.2184727 36815759

[phy270050-bib-0031] Pella, E. , Boutou, A. , Boulmpou, A. , Papadopoulos, C. E. , Papagianni, A. , & Sarafidis, P. (2022). Cardiopulmonary exercise testing (CPET) in patients with end‐stage kidney disease (ESKD): Principles, methodology and clinical applications of the optimal tool for exercise tolerance evaluation. Nephrology, Dialysis, Transplantation, 37(12), 2335–2350. 10.1093/ndt/gfab150 33823012

[phy270050-bib-0032] Pella, E. , Theodorakopoulou, M. P. , Boutou, A. K. , Alexandrou, M. E. , Bakaloudi, D. R. , Sarridou, D. , Boulmpou, A. , Papadopoulos, C. , Papagianni, A. , & Sarafidis, P. (2022). Cardiopulmonary reserve examined with cardiopulmonary exercise testing in individuals with chronic kidney disease: A systematic review and meta‐analysis. Annals of Physical and Rehabilitation Medicine, 65(5), 101588. 34634515 10.1016/j.rehab.2021.101588

[phy270050-bib-0033] Ramírez‐Vélez, R. , Pérez‐Sousa, M. A. , Venegas‐Sanabria, L. C. , Cano‐Gutierrez, C. A. , Hernández‐Quiñonez, P. A. , Rincón‐Pabón, D. , García‐Hermoso, A. , Zambom‐Ferraresi, F. , Sáez de Asteasu, M. L. , & Izquierdo, M. (2020). Normative values for the short physical performance battery (SPPB) and their association with anthropometric variables in older Colombian adults. Frontiers in Medicine, 7, 52. 32154258 10.3389/fmed.2020.00052 PMC7044127

[phy270050-bib-0034] Rikli, R. E. , & Jones, C. J. (1999). Functional fitness normative scores for community‐residing older adults, ages 60‐94. Journal of Aging and Physical Activity, 7(2), 162–181.

[phy270050-bib-0035] Sabatino, A. , Cuppari, L. , Stenvinkel, P. , Lindholm, B. , & Avesani, C. M. (2021). Sarcopenia in chronic kidney disease: What have we learned so far? Journal of Nephrology, 34(4), 1347. 32876940 10.1007/s40620-020-00840-y PMC8357704

[phy270050-bib-0036] Strassmann, A. , Steurer‐Stey, C. , Lana, K. D. , Zoller, M. , Turk, A. J. , Suter, P. , & Puhan, M. A. (2013). Population‐based reference values for the 1‐min sit‐to‐stand test. International Journal of Public Health, 58(6), 949–953. 23974352 10.1007/s00038-013-0504-z

[phy270050-bib-0037] Stray‐Gundersen, J. , Howden, E. J. , Parsons, D. B. , & Thompson, J. R. (2016). Neither hematocrit normalization nor exercise training restores oxygen consumption to Normal levels in hemodialysis patients. Journal of the American Society of Nephrology, 27(12), 3769–3779. 27153927 10.1681/ASN.2015091034 PMC5118480

[phy270050-bib-0038] Susantitaphong, P. , Koulouridis, I. , Balk, E. M. , Madias, N. E. , & Jaber, B. L. (2012). Effect of frequent or extended hemodialysis on cardiovascular parameters: A meta‐analysis. American Journal of Kidney Diseases, 59(5), 689–699. 22370022 10.1053/j.ajkd.2011.12.020 PMC3395217

[phy270050-bib-0039] Theodorakopoulou, M. P. , Zafeiridis, A. , Dipla, K. , Faitatzidou, D. , Koutlas, A. , Alexandrou, M.‐E. , Doumas, M. , Papagianni, A. , & Sarafidis, P. (2023). Muscle oxygenation and microvascular reactivity across different stages of CKD: A near‐infrared spectroscopy study. American Journal of Kidney Diseases: The Official Journal of the National Kidney Foundation, 81, 655–664. 36608922 10.1053/j.ajkd.2022.11.013

[phy270050-bib-0040] Ting, S. M. S. , Hamborg, T. , McGregor, G. , Oxborough, D. , Lim, K. , Koganti, S. , Aldridge, N. , Imray, C. , Bland, R. , Fletcher, S. , Krishnan, N. S. , Higgins, R. M. , Townend, J. , Banerjee, P. , & Zehnder, D. (2015). Reduced cardiovascular Reserve in Chronic Kidney Failure: A matched cohort study. American Journal of Kidney Diseases, 66(2), 274–284. 25900597 10.1053/j.ajkd.2015.02.335

[phy270050-bib-0041] Ting, S. M. S. , Iqbal, H. , Kanji, H. , Hamborg, T. , Aldridge, N. , Krishnan, N. , Imray, C. H. E. , Banerjee, P. , Bland, R. , Higgins, R. , & Zehnder, D. (2014). Functional cardiovascular reserve predicts survival pre‐kidney and post‐kidney transplantation. Journal of the American Society of Nephrology, 25(1), 187–195. 24231666 10.1681/ASN.2013040348 PMC3871777

[phy270050-bib-0042] Vasunilashorn, S. , Coppin, A. K. , Patel, K. V. , Lauretani, F. , Ferrucci, L. , Bandinelli, S. , & Guralnik, J. M. (2009). Use of the short physical performance battery score to predict loss of ability to walk 400 meters: Analysis from the InCHIANTI study. The journals of gerontology, 64A(2), 223–229. 10.1093/gerona/gln022 PMC2655026 19182232

[phy270050-bib-0043] Vogt, B. P. , Borges, M. C. C. , De, G. C. R. , & Caramori, J. C. T. (2016). Handgrip strength is an independent predictor of all‐cause mortality in maintenance dialysis patients. Clinical Nutrition, 35(6), 1429–1433. 27083497 10.1016/j.clnu.2016.03.020

[phy270050-bib-0044] Wang, A. Y. , Sherrington, C. , Toyama, T. , Gallagher, M. P. , Cass, A. , Hirakawa, Y. , Li, Q. , Sukkar, L. , Snelling, P. , & Jardine, M. J. (2017). Muscle strength, mobility, quality of life and falls in patients on maintenance haemodialysis: A prospective study. Nephrology, 22(3), 220–227. 10.1111/nep.12749 26890468

[phy270050-bib-0045] Wang, Y. C. , Bohannon, R. W. , Li, X. , Sindhu, B. , & Kapellusch, J. (2018). Hand‐grip strength: Normative reference values and equations for individuals 18 to 85 years of age residing in the United States. The Journal of Orthopaedic and Sports Physical Therapy, 48(9), 685–693. 10.2519/jospt.2018.7851 29792107

[phy270050-bib-0046] Wasserman, K. , Hansen, J. , Sue, D. Y. , Stringer, W. , & Whipp, B. J. (2004). Principles of exercise testing and interpretation: Pathophysiology and clinical applications. Balt Maryl, 1, 1–180.

[phy270050-bib-0047] Wasserman, K. , & Whipp, B. J. (1975). Exercise physiology in health and disease. The American Review of Respiratory Disease, 112(2), 219–249. 10.1164/arrd.1975.112.2.219 239617

[phy270050-bib-0048] Wilkinson, T. J. , Gabrys, I. , Lightfoot, C. J. , Lambert, K. , Baker, L. A. , Billany, R. E. , Kanavaki, A. , Palmer, J. , Robinson, K. A. , Nixon, D. , Watson, E. L. , & Smith, A. C. (2022). A systematic review of handgrip strength measurement in clinical and epidemiological studies of kidney disease: Toward a standardized approach. Journal of Renal Nutrition, 32(4), 371–381. 10.1053/j.jrn.2021.06.005 34294555

[phy270050-bib-0049] Wilkinson, T. J. , Miksza, J. , Yates, T. , Lightfoot, C. J. , Baker, L. A. , Watson, E. L. , Zaccardi, F. , & Smith, A. C. (2021). Association of sarcopenia with mortality and end‐stage renal disease in those with chronic kidney disease: A UK biobank study. Journal of cachexia, sarcopenia and muscle, 12(3), 586–598. 10.1002/jcsm.12705 33949807 PMC8200422

[phy270050-bib-0050] Wilkinson, T. J. , White, A. E. M. , Daniel, N. G. D. , Douglas, G. W. , Nixon, D. G. D. , Gould, D. W. , Watson, E. L. , & Smith, A. C. (2019). Characterising skeletal muscle haemoglobin saturation during exercise using near‐infrared spectroscopy in chronic kidney disease. Clinical and Experimental Nephrology, 23, 32–42. 10.1007/s10157-018-1612-0 29961156 PMC6344386

